# Feasibility of transcontinental coaching of complex robotic thoracic surgical procedures: A bronchial anastomosis on a 3-dimensional model

**DOI:** 10.1016/j.xjtc.2025.01.014

**Published:** 2025-01-25

**Authors:** Kohei Hashimoto, Yoshifumi Hirata, Takashi Nozawa, Jun Yamaguchi, Hiroshi Fukuhara, Haruhiko Kondo, Kazuhiro Yasufuku

**Affiliations:** a Division of Thoracic Surgery, Kyorin University, Tokyo, Japan; b Department of Clinical Engineering, Kyorin University Hospital, Tokyo, Japan; c Department of Urology, Kyorin University, Tokyo, Japan; d Division of Thoracic Surgery, Toronto General Hospital, University Health Network, University of Toronto, Toronto, Canada

**Figure:**
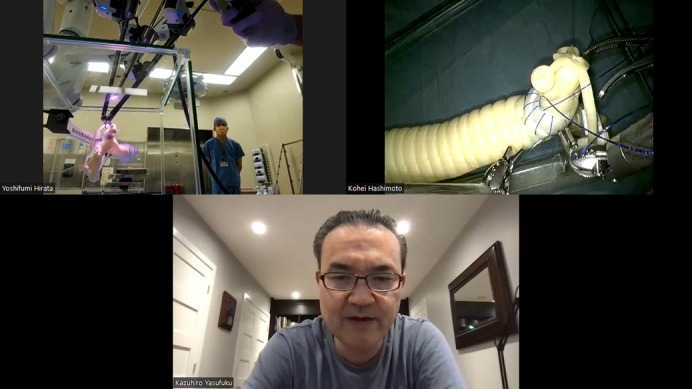
Transcontinental proctoring of robotic sleeve resection through a web-based platform.

**Central Message**

Transcontinental coaching of robotic bronchoplasty using publicly available software was feasible. This implies how surgical robots can enhance surgical education by experts regardless of borders.

Surgical robots for visceral surgery were originally designed to allow surgeons to operate remotely on battlefield wounds.^1^ In 2001, the first remote robotic procedure trial, called the Lindburg Operation, was performed. The patient was located in Strasbourg, France, while the surgeon controlled the robot from New York in the United States.^2^ More recently, the robotic platform has been developed as an advanced tool for endoscopic surgery. Currently, remote robotic procedures and education are being revisited to optimize the limited resources of expert robotic surgeons.^3^^,^^4^ Several software platforms or products that enable remote education, such as Intuitive Hub (Intuitive), have been introduced. However, how to enhance robotic training using these communication tools has not been well described in the literature. We became familiar with web-based communication tools after the COVID-19 pandemic. This tool opened global networking opportunities. We hypothesized that coaching of complex robotic procedures across the continents using publicly available web-based communication software (Zoom; Zoom Communications Inc) is feasible and can help extend the knowledge and skills of a limited number of experts to the world.

## Methods

### Three-Dimensional Airway Model and Surgical Instruments

**Figure 1:**
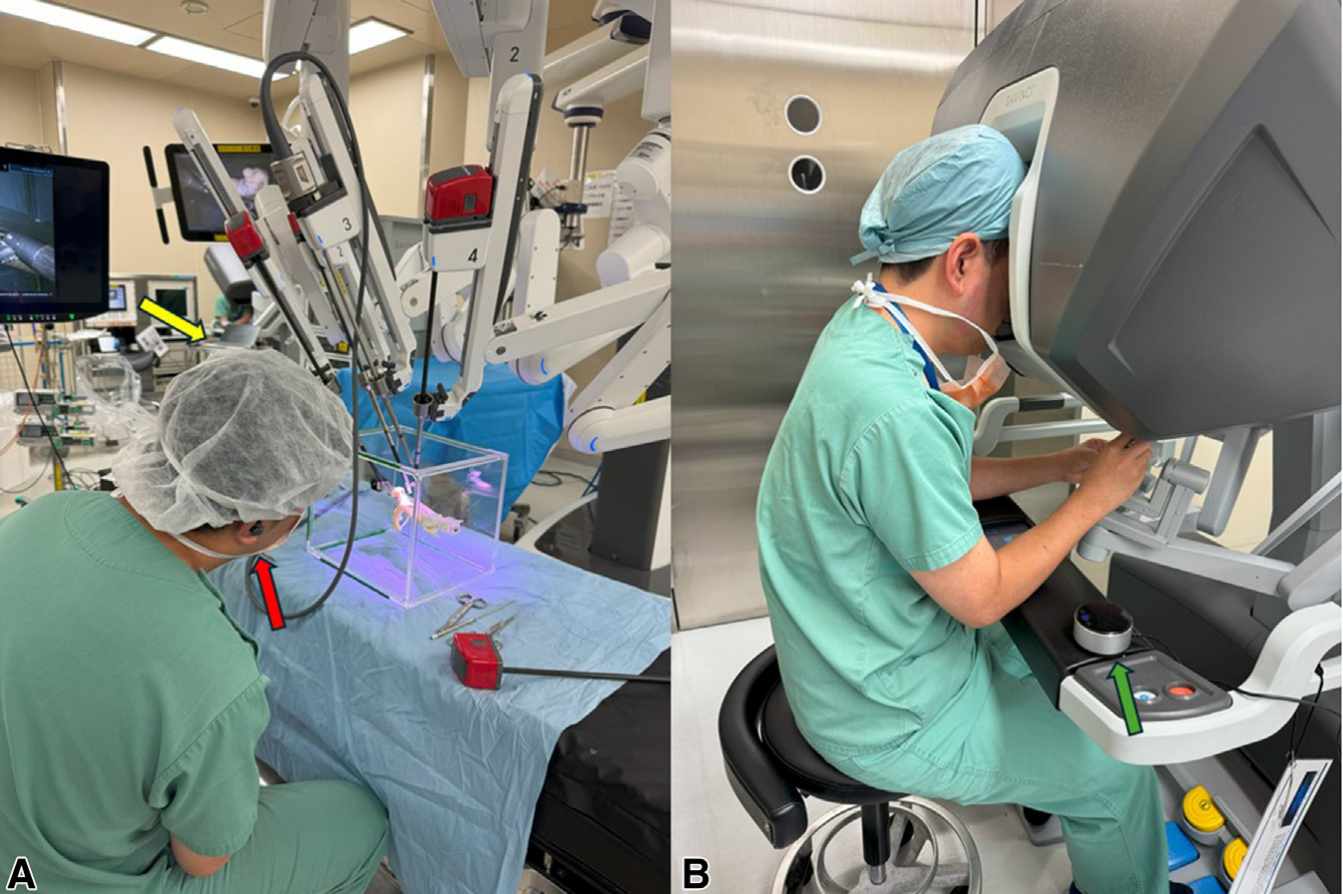
Setup of robot and 3-dimensional airway model for transcontinental robotic proctoring. A, Bedside setting with an assistant. The red arrow indicates a wireless earpiece and the *yellow arrow* indicates a laptop computer. B, Console setting with an operator. The *green arrow* indicates a speakerphone.

The bronchial anastomosis was performed on a 3-dimensional (3D) airway model previously developed by us.^5^ The da Vinci Xi surgical system (Intuitive) was used (Figure 1, *A*). Two large needle drivers were used in each hand, and a Cadiere forceps was used for the retraction arm. Two 4-0 barbed sutures with 22-cm long thread and 22-mm 1/2 atraumatic needle (StrataFix; Ethicon) were used for the running suture. Institutional review board approval was waved because no patient was involved in the study.

### Setup of the Web-Based Communication Platform

**Figure 2:**
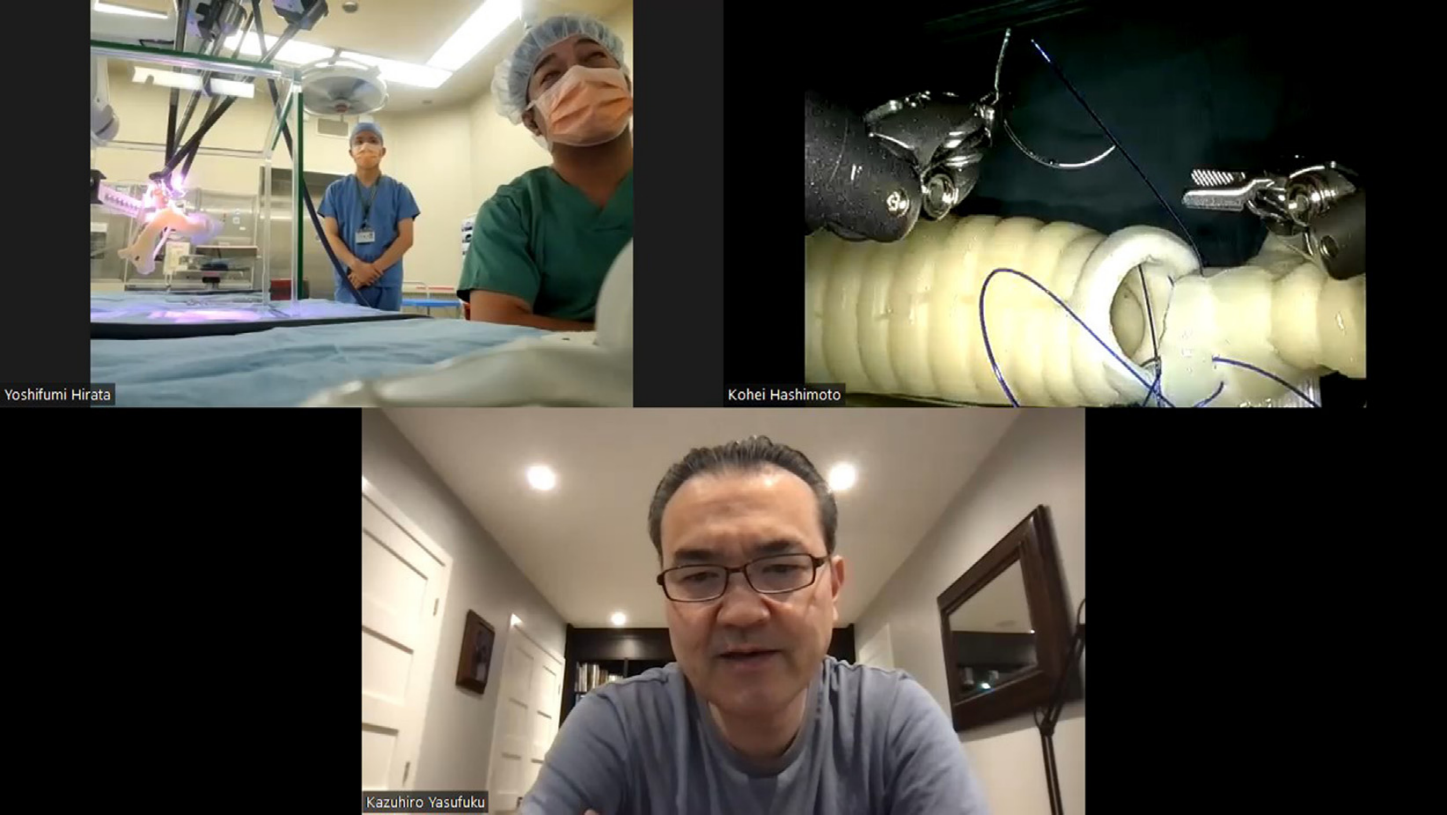
View of transcontinental robotic proctoring between Japan and Canada.

A laptop notebook computer (IdeaPad Slim 550, 16 GB RAM, AMD Ryzen 7 4700U processor, Octo-Core 2 GHz, integrated AMD Radeon graphics; Lenovo) was used with the Zoom platform (workplace version 6.0.11). A wired local area network was used for internet connection. Images were extracted from the vision cart using a digital visual interface -digital (DVD-D) to high-definition multimedia interface (HDMI) adapter (VV-HDDV050CA-B; VodaView) and then transferred to the laptop computer using a capture board with high-definition multimedia interface to universal serial bus type C (USB-C) conversion (Hu_04A; C-Amour). In this way, Zoom can incorporate the console view by selecting the capture board view in “Camera Function.” The computer was connected to a speakerphone (TSK95 K; TAMA Electrics Industry Corp) placed on the surgeon's console (Figure 1, *B*). The speaker function on the patient cart was turned off to avoid noise, and the bedside assistant used a wireless earpiece to hear the surgeon's voice connected to another smartphone on Zoom (Figure 1, *A*). A Board-certified thoracic surgeon (K.H.) performed a right upper sleeve lobectomy simulated on the 3D model in a real operating room at Kyorin University Hospital in Tokyo, Japan, while a robotic thoracic surgery expert (K.Y.) in Toronto, Ontario, Canada, coached through the Zoom platform. Verbal technical instructions on how to improve the anastomosis were given. The operating surgeon is very experienced in open sleeve resection but does not have experience in robotic sleeve resection. Verbal technical instructions on how to improve anastomosis were given. The recording function (speakerphone setting) was used in Zoom (Figure 2).

## Results

Measured internet transmission speeds in Tokyo were approximately 100 Mb/second uplink and 95 Mb/second downlink. In Toronto, these were approximately 53 Mb/second uplink and 228 Mb/second downlink. The measured quality of the robotic surgery console view in the coach's Zoom was 7 to 10 milliseconds latency, 0 to 1 milliseconds jitter, 0.0% packet loss, 640 × 360 resolution, and 20 to 24 frames per second. With this setup, 2 surgeons could communicate simultaneously and share the console view with no noticeable delay in voice or view (Video 1). Occasional audio dropouts were observed. This appeared to be a microphone problem, not a connection problem because there was no video interruption. The surgeon in Japan was able to hear the instructions from Canada through a speaker and was able to immediately implement the advised techniques. The anastomosis was successfully completed on the 3D airway model with ongoing instruction. It took 23 minutes. Throughout the procedure, the transmitted console view appeared smooth enough to the coaching surgeon in Canada.

## Discussion

In this study, we have shown the feasibility of coaching of robotic procedures through a publicly available web-based networking tool, even across the continents. Surgical robotics are being implemented by multiple companies in multiple countries, and new surgical procedures are being developed as robotic technologies advance. However, because of population and economy disparities, the availability of local expertise varies widely. A wider application of this web-based tool could connect robotic surgeons worldwide, thereby enhancing teleproctoring. Intuitive is releasing the latest version of its platform (DV5, fifth generation) with teleproctoring functionality. This functionality may be implemented on other companies' platforms in the future and may be the norm for next-generation robotic platforms. Theoretically, it is also possible to use Zoom to transmit the surgical view of video-assisted procedures and even open procedures for teleproctoring, which could be another benefit of our methods.

This feasibility study has limitations. First, we could use this system without ethical review because no clinical material was used. Ethical approval as well as web security will need to be obtained to ensure patient privacy and safety when translating this into clinical settings. According to Zoom (https://support.zoom.com/hc/en/article?id=zm_kb&sysparm_article=KB0067751), its service can be Health Insurance Portability and Accountability Act compliant in the United States with certain conditions. Second, internet speed and regulation may differ significantly between counties or hospitals. One needs to check his or her own local network environment to ensure the quality of communication. Note that the internet speed (100 Mb/second) used in our study is not very fast in modern systems (home internet speed in North America can start at 200 Mb/second). This level of speed should be achievable in many areas. The minimum internet speed requirement for a stable Zoom connection is about 2.0 Mb/second for a gallery view conversation used in this setting according to the Zoom home page. Third, the pointer function available during onsite Dual console proctoring was not included. The lack of this feature may have limited the quality of the coaching in our setting; for example, where exactly to put the needle. This may improve with technological advances in the near future. Nevertheless, we recognize that remote coaching is not equivalent to onsite coaching, but we believe that this remote method helps to transfer knowledge from experts to widespread robotic surgeons, especially in areas with limited local experts.

## Conclusions

In our study, transcontinental coaching of a robotic bronchial sleeve resection procedure was feasible using a publicly available web-based platform. We believe that this method will open new possibilities for how surgical robots can enhance the coaching of complex surgical procedures by the best surgeons regardless of borders.

### Webcast

You can watch a Webcast of this AATS meeting presentation by going to: xxx.

## Conflict of Interest Statement

The authors reported no conflicts of interest.

The *Journal* policy requires editors and reviewers to disclose conflicts of interest and to decline handling or reviewing manuscripts for which they may have a conflict of interest. The editors and reviewers of this article have no conflicts of interest.

## Supplementary Data

Video 1 Recorded view of transcontinental proctoring of a right upper sleeve resection procedure on the 3-dimensional airway model. Video available at: https://www.jtcvs.org/article/S2666-2507(25)00050-1/fulltext.
