# Supplementary figures and images for: Feasibility of transcontinental coaching of complex robotic thoracic surgical procedures: A bronchial anastomosis on a 3-dimensional model
Source: JTCVS Tech. 2025 Jan 25;32:209–11. doi: 10.1016/j.xjtc.2025.01.014 (PMC12347286; doi:10.1016/j.xjtc.2025.01.014)

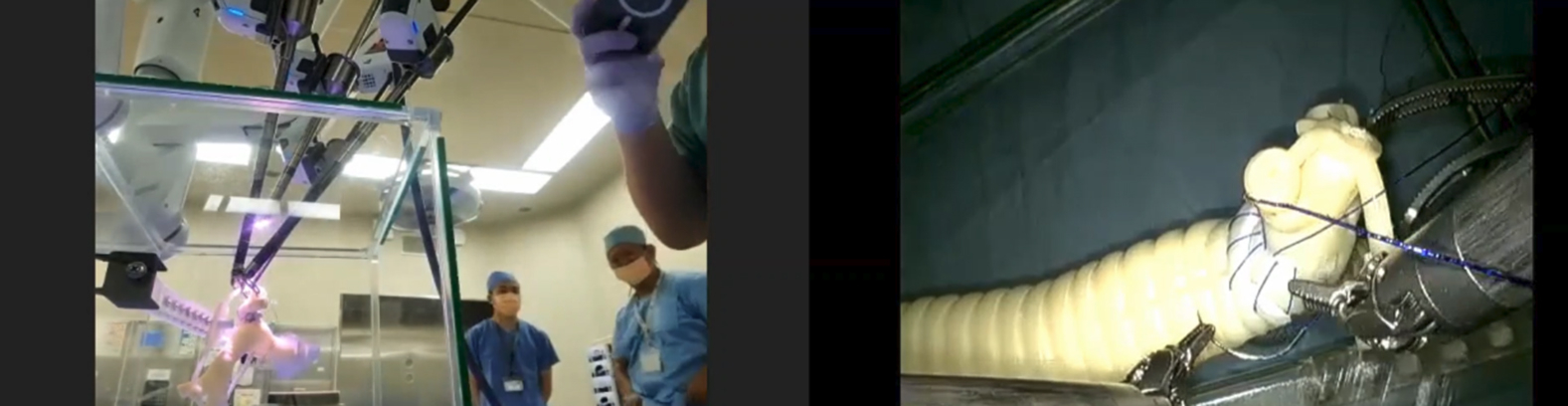

Supplement: Video 1 — Recorded view of transcontinental proctoring of a right upper sleeve resection procedure on the 3-dimensional airway model. Video available at: https://www.jtcvs.org/article/S2666-2507(25)00050-1/fulltext. [file fx2.jpg]
